# Deregulated hypoxic response in myeloid cells: A model for high‐altitude pulmonary oedema (HAPE)

**DOI:** 10.1111/apha.13461

**Published:** 2020-03-16

**Authors:** Milos Gojkovic, Gabriella S. Darmasaputra, Pedro Veliça, Helene Rundqvist, Randall S. Johnson

**Affiliations:** ^1^ Department of Cell and Molecular Biology Karolinska Institute Stockholm Sweden; ^2^ University of Utrecht Faculty of Medicine ‐ Cancer Biology Utrecht Netherlands; ^3^ Department of Physiology and Pharmacology Karolinska Institute Stockholm Sweden; ^4^ Department of Physiology Development and Neuroscience University of Cambridge Cambridge UK

**Keywords:** HAPE model, inflammation, myeloid hypoxia, permeability, pulmonary oedema, VHL

## Abstract

**Aim:**

High‐altitude pulmonary oedema (HAPE) is a non‐cardiogenic pulmonary oedema that can occur during rapid ascent to a high‐altitude environment. Classically, HAPE has been described as a condition resulting from a combination of pulmonary vasoconstriction and hypertension. Inflammation has been described as important in HAPE, although as a side effect of pulmonary oedema rather than as a causative factor. In this study, we aim to understand the role of hypoxic response in myeloid cells and its involvement in pathogenesis of HAPE.

**Methods:**

We have generated a conditional deletion in mice of the von Hippel‐Lindau factor (VHL) in myeloid cells to determine the effect of a deregulated hypoxic response in pulmonary oedema.

**Results:**

The deletion of VHL in pulmonary myeloid cells gave rise to pulmonary oedema, increased pulmonary vascular permeability and reduced performance during exertion. These changes were accompanied by reduced stroke volume in the left ventricle.

**Conclusion:**

In this model, we show that a deregulated myeloid cell hypoxic response can trigger some of the most important symptoms of HAPE, and thus mice with a deletion of VHL in the myeloid lineage can function as a model of HAPE.

## 1 INTRODUCTION

Pulmonary oedema is a condition defined by an increased amount of extravascular fluid in the lung.^1^ Normally fluid levels in the alveolar space are tightly regulated and balanced by active ion transport in the alveolar epithelium.^2^ Disturbance in this homeostatic regulation of alveolar fluid can lead to pulmonary oedema,^3^ whose causes can be divided into two main categories: cardiogenic, generally from hypertension in the pulmonary circulation; and non‐cardiogenic, where inflammation, tissue damage or cancer leads to disturbances in vascular permeability.^4^

One of the more commonly known syndromes involving pulmonary oedemas is high‐altitude pulmonary oedema (HAPE), one of the non‐cardiogenic pulmonary oedema.^5^ HAPE occurs in certain individuals after a rapid ascent to high altitude, and can be life‐threatening.^6^ Early signs of HAPE are cough, dyspnoea and a reduced capacity for exertion, which then progresses to breathlessness at rest and orthopnoea.^7^ The main driver of HAPE has generally been considered to be pulmonary vasoconstriction and hypertension driving the accumulation of alveolar fluid.^6^, ^7^ Currently, treatment for HAPE consists of vasodilators, corticosteroids, supplementation of oxygen and descending to lower altitude.^8^ There is currently little consensus on whether HAPE should be considered a non‐inflammatory condition or not.^6^, ^9^

In one randomized clinical trial, subjects with a history of HAPE were shown to resist development of pulmonary oedema when treated with dexamethasone, an anti‐inflammatory corticosteroid.^10^ Other studies have shown that individuals with HAPE have increased cell counts in bronchoalveolar lavage (BAL), indicating inflammation and detectable levels of inflammatory cytokines such as IL‐1, IL‐6 and TNFa.^11^ Further evidence that inflammation and HAPE are closely connected is the finding that preexisting inflammation is a risk factor in HAPE,^12^ and that individuals with HLA‐DR6 and HLA‐DQ4 haplotypes are at increased risk of developing HAPE.^13^ Increased levels of the hypoxia‐induced, myloid‐derived, cytokines MCP‐1 and MIP‐1 have been found in blood plasma of HAPE‐sensitive individuals.^14^ The importance of inflammation in HAPE has also been shown in animal models. Mice exposed to hypoxia show early recruitment of macrophages and monocytes to pulmonary tissue; this is an essential response for hypoxia‐induced pulmonary remodelling.^15^

Hypoxia inducible factor 1 and 2 (heterodimers with the oxygen‐labile components HIF1a and HIF2a) constitutively expressed transcriptional regulators responsible for transcription of multiple genes required for cellular adaptation to hypoxia. Primary regulation of HIF1 and HIF2 activity occurs through proteasomal degradation via ubiquitination initiated by the von Hippel‐Lindau protein (VHL).^16^ Mutations deregulating HIF1a/HIF2a‐Pathway have been observed among populations in high‐altitude environments^17^, ^18^ and may be adaptations to chronic exposure to hypoxia. Additionally, HIF1a and HIF2a activity have been shown to play an important role in several myeloid functions and inflammatory responses.^19^, ^20^, ^21^, ^22^, ^23^ Hypoxia, vascular permeability, pulmonary oedema and inflammation seem to be processes partially or primarily regulated by HIF1a and HIF2a pathways in myeloid cells.

We hypothesized that myeloid cells and their responses to hypoxia likely play a significant role in HAPE, and thus decided to explore the role of myeloid HIF1a and HIF2a in HAPE.

## 2 RESULTS

In order to further understand the role of myeloid cells in pulmonary hypoxia, we generated a mouse model lacking negative regulation of HIF1a and HIF2a, mimicking a constant hypoxia response. To accomplish this, we carried out studies with mice homozygous for a conditional mutation in the VHL gene, (VHL^fl/fl^ carrying a myeloid‐specific cre recombinase allele LysM^Cre+^).^24^

Mice homozygous for the conditional VHL allele and the myeloid‐specific cre recombinase will have increased expression of HIF1α and HIF2α also under high oxygen conditions which will alter the expression of several genes. We have confirmed this in frozen lung sections in Figure S1A for HIF1a and Figure S2A for HIF2a and in bone marrow‐derived macrophages (BMDM) in Figure S1C.

As lungs have a specific response to hypoxia, resulting in vasoconstriction and in severe situations also oedema, we wanted to understand the role of myeloid cells in this response. Initially, we characterized the gene expression of several HIF1a and HIF2a targets in the lungs.

**FIGURE 1:**
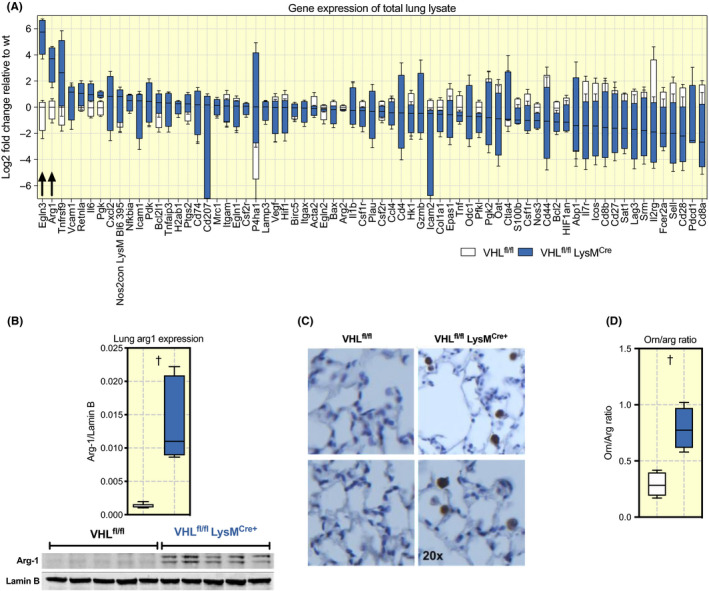
Mice lacking myeloid VHL have an increased expression of Egln3 and Arg‐1 in the whole Lung. A, cDNA from whole lung extract was used to determine different transcription levels of genes presented in the graph, all values are shown in Log2 fold change relative to VHL^fl/fl^. B, Increased expression of Arg‐1 was confirmed using Western blot on whole lung lysates, (C) immunohistochemistry and Arg activity was confirmedby (D) ornithine/ arginine ratio in pulmonary extracts by UHPLC‐QqQ‐MSMS. Data presented as Tukey boxplots, arrow or ^†^ *P* < .01, ns = not significant. Statistical analysis was performed with unpaired T test, n = 6‐11 mice per group aged between 10 and 16 weeks. VHL, von Hippel‐Lindau

RNA from whole mouse lungs from mutant VHL^fl/fl^ mice and control VHL^fl/f^ LysM^Cre+^ mice were screened for expression of genes involved in HIF signalling, pulmonary function and inflammatory response. We found that the Egln3 and Arg‐1 genes were expressed at high levels in mutant mice compared to control mice (Figure 1A). Arg‐1 expression was confirmed to be elevated at protein level in the lungs of mutant mice (Figure 1B). Histological assessment revealed that most Arg‐1+ cells could be found in the alveolar space (Figure 1C). As the primary substrate for the product of the Arg‐1 gene, the Arginase‐1 enzyme, is the amino acid arginine, we used mass spectrometry to determine the levels of arginase metabolites in the lungs. These analyses showed an increased Ornithine/Arginine ratio, demonstrating increased arginase activity in the lungs (Figure 1D).

**FIGURE 2:**
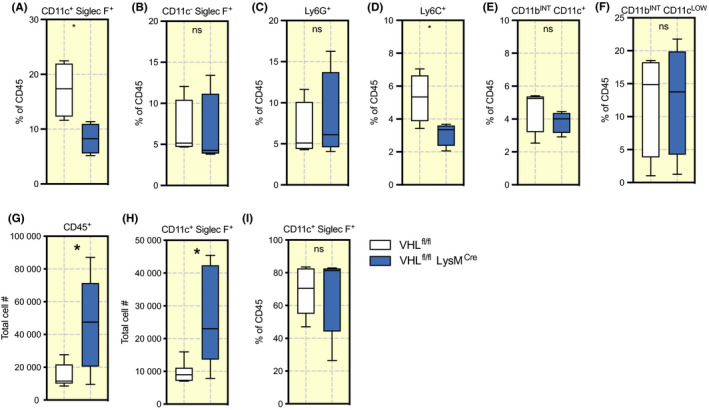
Immunological composition of pulmonary myeloid populations. Comparison of cell frequencies normalized to leukocytes (CD45^+^) in single‐cell suspension of the total lung (A) alveolar macrophages as CD11c^+^ Siglec F^+^, (B) eosinophils as CD11c^+^ Siglec F^−^, (C) neutrophils as Ly6G^+^, (D) monocytes as Ly6c^+^, and mononuclear myeloid cells as (E) with high CD11b expression (CD11b^hi^ CD11c^+^) and intermediate expression (CD11b^INT^ CD11c^−^), (F) followed by total cell number in BAL on (G) Leukocytes, CD45^+^, (I) Alveolar macrophages, and (I) their frequencies. Data presented as Tukey boxplots, *P < .05, ns = not significant. Statistical analysis was performed with unpaired T test, n = 4‐5 mice per group aged between 10 and 16 weeks. BAL, bronchoalveolar lavage

We then characterized single‐cell suspensions from perfused mouse lungs to remove the cells of the blood stream. We found that VHL^fl/fl^ LysM^Cre+^‐mutant mice have lower numbers of monocytes (Ly6C^+^ CD11b^+^) and alveolar macrophages (SiglecF^+^ CD11c^+^), however, other myeloid populations remained unchanged (Figure 2A‐F and Figure S2A‐H). Immune cell populations found in BAL showed a higher number of CD45^+^ cells (Figure 2G) and alveolar macrophages (Figure 2H,I) with no additional changes in other alveolar population numbers (Figure S3A‐H). The differences in alveolar populations versus the whole lung populations could be because of an increased amount of alveolar fluid in VHL^fl/fl^ LysM^Cre+^, making the alveolar cells more accessible during collection of BAL.

**FIGURE 3:**
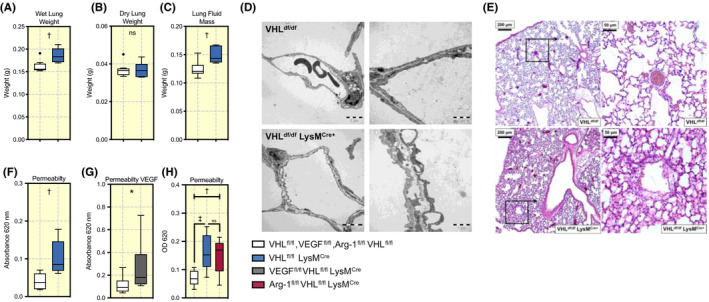
Deletion of myeloid VHL results in pulmonary oedema endothelial fenestration and increased pulmonary permeability. Lungs were collected from wild type floxed mice (VHL^fl/fl^) and mice lacking myeloid VHL (VHL^fl/fl^ LysM^Cre+^) aged between 10 and 12 weeks. A, Total lung weight (in grams), (B) the lung weight post dehydration in 60°C for 3 days, (C) lung fluid mass determined by reduction of dry lung weight from total lung weight. D, Pictures acquired by electron microscopy and (E) H&E staining comparing VHL^fl/fl^ mice with VHL^fl/fl^ LysM^Cre+^ mice. F, 1% Evans blue in PBS was administered i.v to VHL^fl/fl^ and VHL^fl/fl^ LysM^Cre+^ mice respectively. One hour later BAL was collected and permeability determined by measurement of Evans blue content at absorbance 620 nm, (G) Same procedure performed in VEGF^fl/fl^ VHL^fl/fl^ LysM^cre+^ mice, and (H) in Arg‐1^fl/fl^ VHL^fl/fl^ LysM ^Cre+^ mice. Data presented as Tukey boxplots, *P < .05, ^†^ *P* < .01, ^‡^ *P* < .001, ns = not significant. Statistical analysis was performed with unpaired T test, n = 7‐8 mice per group. BAL, bronchoalveolar lavage; VHL, von Hippel‐Lindau

Our following observations of mutants lacking myeloid VHL showed that wet lung weight was increased in the mutant mice (Figure 3A), with no difference in dry lung weight (Figure 3B), thus an increased lung fluid mass (Figure 3C). Total body weight between the two groups was not different (Figure S4A), however, lung/body weight ratio was increased in VHL^fl/fl^LysM^Cre+^ mutant mice (Figure S4B). Thus, loss of VHL in myeloid cells gives rise to a detectable pulmonary oedema in mutants.

To learn whether if the observed pulmonary oedema might be caused by any structural differences in the pulmonary tissue, a qualitative assessment by electron microscopy was performed, revealing signs of endothelial cell fenestrations (Figure 3D) with no other vascular remodelling being observed in VHL^fl/fl^ LysM^Cre+^ mice. Histological assessment also showed signs of increased eosin stain in the alveolar space of VHL^fl/fl^ LysM^Cre+^ mice (Figure 3E) This indicates that the observed phenotype might be because of an increased pulmonary endothelial permeability. This hypothesis was tested using an Evans blue‐based permeability assay, in which mice were injected with dye intravenously and pulmonary permeability was determined by measurement of dye levels in BAL 1‐hour post‐injection. VHL^fl/fl^ LysM^Cre+^‐mutant mice showed increased levels of dye in BAL, demonstrating increased pulmonary permeability (Figure 3F). The angiogenic and permeability factor vascular endothelial growth factor‐A (VEGF‐A) is a known HIF target.^16^ To test the role of VEGF‐A in this model, we generated a myeloid‐specific double deletion mouse strain for both VEGF‐A and VHL, so as to test whether the permeability and oedema seen in VHL mutants is also present in mice lacking both genes in the myeloid lineage; however, the loss of both VEGF‐A and VHL still resulted in a significant degree of increase pulmonary vascular permeability relative to controls (Figure 3G). Observing that Arg‐1 expression and activity was increased in VHL^fl/fl^ LysM^Cre+^ mice, we generated a double knockout Arg‐1^fl/fl^ VHL^fl/fl^ LysM^Cre+^ mice to find out if Arg‐1 was responsible for increased permeability. The level of permeability in Arg‐1^fl/fl^ VHL^fl/fl^ LysM^Cre+^ was significantly higher than Arg‐1^fl/fl^ VHL^fl/fl^ and similar to VHL^fl/fl^ LysM^Cre+^ mice (Figure 3H).

**FIGURE 4:**
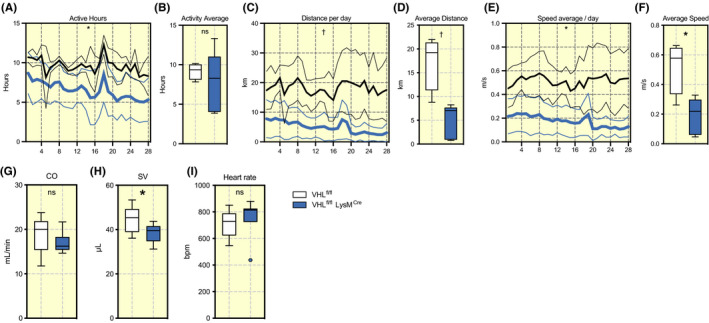
Deleted myeloid VHL results in reduced physical performance and decreased stroke volume. Mice were housed in pairs with monitoring running wheels for 4 weeks. A, Daily activity, number of hours spent on the wheel, (B) summarized as 28‐day average (total average), (C) daily distance, (D) total average of distance, (E) speed and (F) total speed average. Echocardiography data on anaesthetized and unchallanged mice of the left ventricle in SAX‐M mode showing (G) CO, (H) Stroke volume, (I) heart rate. Data presented as standard error and mean and Tukey boxplots, *P < .05, ^†^ *P* < .01, ns = not significant. Statistical analysis was performed with mixed effect analysis and unpaired T test, n = 2 mice/wheel total 8‐10 mice/group. CO, cardiac output; VHL, von Hippel‐Lindau

Pulmonary oedema is known to have physiological consequences for exertion and cardiovascular function generally. We performed a series of physiological tests to confirm if this is the case for this model. Mice were placed in cages with running wheels with activity tracking for 28 days (post 4 day period of acclimatization period). As shown in Figure 4A,B the total activity per day between VHL^fl/fl^ LysM^Cre+^‐mutant mice, and VHL^fl/fl^ wild type control mice is slightly reduced. However, the VHL^fl/fl^ LysM^Cre+^‐mutant mice cover less distance in total (Figure 4C,D), and run at a slower pace overall, as seen in Figure 4E,F. Thus, mutant animals have the same tendency to attempt exercise, but a reduced capacity for voluntary running.

To fully understand the underlying mechanism behind the decreased physical performance in VHL^fl/fl^ LysM^Cre+^ mice and to investigate any cardiogenic effects, tail cuff blood pressure, echocardiography and electrocardiography (ECG) was carried out. Echocardiography data demonstrated no difference in cardiac output (Figure 4G) and reduced stroke volumes (Figure 4H) and confirmed with reduced tail blood volume (Figure S5A), and a trend towards higher heart rate can be seen (Figure 4I). Mean arterial pressures were not different between the two groups (Figure S5B) and this was also the case for diastolic and systolic pressures (Figure S5C,D). However, we observed a trend to small reduction in average blood flow (Figure S5E). Further on, short axis and motion view (SAX‐M mode) of the heart in echocardiography did not show any difference in other cardiac parameters (Figure S6A‐E).

Both tail cuff and echocardiology data confirm that mice have reduced stroke volumes, possibly resulting in reduced oxygen carrying capacity and reduced perfusion of peripheral tissues, thereby inhibiting high intensity exertion. ECG data did not show any clear abnormalities (Figure S5F‐Q), except for a significantly reduced QTc dispersion in mutant VHL^fl/fl^ LysM^Cre+^ mice Figure S5P). The reduced stroke volume could possibly be explained as a loss of volume in the pulmonary circulation, resulting in less volume returning from the lungs to the heart. A series of other physiological tests have been performed, showing no difference in O_2_ Consumption (Figure S6F) or CO_2_ production (Figure S6G) and showing no metabolic differences between the groups metabolism at baseline. Other pulmonary functions tested, including sensitivity to methacholine (Figure S7A‐I) or to a short acute hypoxia challenge (Figure S8A‐I) demonstrated no differences between mutants and wild type controls.

In an attempt to gain a deeper understanding of the mechanisms behind our observed phenotypes, we performed a single read RNA sequencing on the whole lungs of VHL^fl/fl^ LysM^Cre+^ mice (with VHL^fl/fl^ as our wildtype control). Knocking out myeloid VHL resulted in altered expression of 436 pulmonary genes, having 44 genes significantly downregulated and 392 upregulated (Figure S9B). Most of these genes were found to be involved in processes such as inflammatory response, metabolic processes and ammonium transmembrane transport (Figure S9C), which is also represented and confirmed by functions of the 10 most downregulated or upregulated genes (Figure S9D).

**FIGURE 5:**
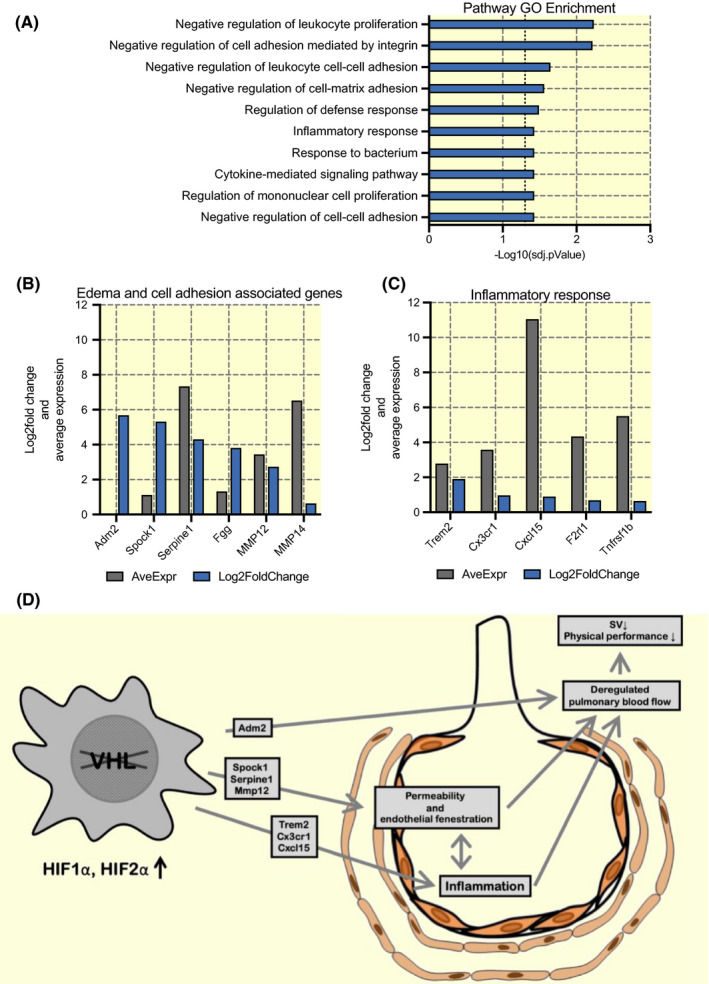
Deleted myeloid VHL results in upregulation of genes involved in cellular adhesion and inflammatory response. RNA sequencing performed on murine female lungs at age of 11 weeks. A, GO pathway enrichment of interest, adjusted *P*‐value demonstrated as negative log10. B, Upregulated genes previously known to be associated with pulmonary oedemas and cellular adhesion and (C) most commonly occurring genes in inflammatory associated pathway enrichments. (D) A model for the role of myeloid HIF in HAPE. Log2fold change is Log2 value of the relative change in VHL^fl/fl^ LysM^Cre+^ mice compared to VHL^fl/fl^, Average Expression is the log 2 of the counts per million of the gene over all samples. n = 4, adjusted *P* values were calculated by false discovery rate test. VHL, von Hippel‐Lindau

Gene Ontology enrichment analysis resulted in positive enrichment for several pathways involved in negative regulation of leukocyte proliferation, cell adhesion and inflammatory pathways (Figure 5A). Genes such as Spock1, Serpine1, Fgg, MMP12 and MMP14 (Figure 5B) that are involved in cellular adhesion have a major increase in expression in VHL^fl/fl^ LysM^Cre+^ compared to VHL^fl/fl^ or are highly abundant in VHL^fl/fl^ LysM^Cre+^, while genes that are part of the inflammatory response and occur in commonly in GO terms that are involved into responses of the immune system such as Trem2, Cx3cr1m Cxcl15, F2rl1 and Tnfrs1b are upregulated in VHL^fl/fl^ LysM^Cre+^ mice and high in abundance (Figure 5C).

## 3 DISCUSSION

Our results demonstrate that deleting myeloid VHL results in pulmonary oedema, increased endothelial cell fenestration and an at least partially VEGF‐A‐independent increase in pulmonary permeability. These results suggest an important role for the myeloid cell in hypoxia‐induced pulmonary permeability.

HAPE leads to reduced physical performance,^7^ and our results indicate a decreased exercise performance rate in VHL^fl/fl^ LysM^Cre+^‐mutant mice, with major reductions in covered distance and running pace. A likely explanation underlying these observations could be the reduced stroke volume observed both by tail cuff measurement and echocardiography, resulting in reduced oxygen carrying capacity and blood flow to peripheral tissues. With unchanged heart rates, blood pressures and ejection fractions ruling out any cardiological complications, a possible explanation behind loss of stroke volume could be dependent on increased blood flow resistance in the pulmonary capillary system because of endothelial fenestration. Another possible explanation could be that the loss of blood volume is caused by the increased pulmonary permeability in VHL^fl/fl^ LysM^Cre+^‐mutant mice, resulting in less blood volume returning to the heart and thus reaching the peripheral tissue.

Comparing the myeloid cell composition in the whole lung versus BAL, we found differing results. In the whole lung, we did not observe any difference in the number of immune cells (CD45^+^) but a reduction in alveolar macrophages and monocytes, whereas in BAL we observed an increased number of immune cells and alveolar macrophages (this has also been observed in humans^11^). This could possibly be explained by the increased amount of alveolar fluid in the mutants.

Transcriptomic analysis revealed more than 300 transcripts being deregulated significantly by deletion of VHL in myeloid cells. Given that our transcriptomics data are for the whole pulmonary tissue, the magnitude of changes confirms that myeloid hypoxia response plays an important role in the pulmonary homeostasis and maintenance. On the most downregulated transcripts in our data set, we found genes that are involved in transmembrane transport of salts and regulation of different inflammatory processes; however, most of these genes show either low abundance or minor changes in expression, suggesting a minimal biological impact on our phenotypic outcome.

A closer look into the profile of genes with upregulated transcription revealed hits that are involved in several inflammatory responses, metabolic processes and most importantly genes involved in negative regulation cellular adhesion to the extracellular matrix. In other tissues, myeloid cells have been shown to be of importance for endothelial permeability.^26^, ^27^

Even though our observations were based on the transcriptional level, we found several candidates that could in a way explain the phenotypic outcome observed in VHL^fl/fl^ LysM^Cre+^ mice. Among the most upregulated genes, we found Egln3 and Arg1 to be present, confirming our earlier observations. *Adm2*, gene encoding for Adrenomedullin 2, a signalling peptide known to cause vasodilation, hypotension, being involved in regulation of endothelial cell permeability and induced by hypoxia.^28^, ^29^, ^30^ These functions suggest that Adrenomedullin 2 could lead to pulmonary vasodilation, causing pulmonary hypotension that results in decreased stroke volume. Further analysis of the data brings forward *Spock1, Serpine1, Mmp12 and Mmp14,* all of which have previously been shown to induce permeability,^31^ contribute to pulmonary oedema,^32^ negatively regulate cellular adhesion to extracellular matrix and impact on endothelial functions^33^, ^34^, ^35^, ^36^ suggesting that these factors may and probably play an important role in permeability, endothelial fenestration and oedema.

Among genes with upregulated expression, we found several genes that are involved in inflammatory response (for example *Cxcr1*,* Trem, Cxcr1, CxcL15, F2rl1* and *Tnfrsf1b*) all of which could be a result of increased extravascular fluid in the alveolar space, or in them self initiate process that leads to pulmonary oedema.

In a collective view, our data seem to suggest multifactorial phenotypes resulting from deletion of VHL in myeloid cells and an increased hypoxic response. It seems very unlikely that any of these phenotypes could be resolved with a single genetic alteration (as our observations in Arg‐1^fl/fl^ VHL^fl/fl^ LysM^Cre+^ and VEGF^fl/fl^ VHL^fl/fl^ LysM^Cre+^ mice confirmed). As illustrated in Figure 5D, deletion of VHL, which leads to stabilization of HIF1a and HIF2a results in increased expression of *Adm2* that in itself can lead to deregulated pulmonary blood flow, resulting in reduced stroke volume and physical performance, while factors such as *Spock1, Serpine1* and *Mmp12* cause endothelial fenestration and increased permeability, contributing to both deregulated blood flow and an increased inflammation. All of this results in a feed forward loop that maintains pulmonary oedema induced by an elevated hypoxic response.

Inflammation has previously been described in the literature as consequence of pulmonary oedema, while our results suggest that inflammation alone, driven by the hypoxic response, can cause pathological events occurring in HAPE.

Given that VHL^fl/fl^ LysM^Cre+^ mice have a hypoxia response‐dependent pulmonary oedema, and that they show reduced physical performance with no clear change in cardiological dysfunction,^6^, ^8^, ^11^ there are a number of aspects of these mice that allow them to be considered as an animal model for HAPE.

## 4 MATERIALS AND METHODS

### 4.1 Animals

VHL^fl/fl^ LysM^Cre+^, VEGF^fl/fl^ VHL^fl/fl^ LysM^Cre+^ and Arg‐1^fl/fl^ VHL^fl/fl^ LysM^Cre+^ mice were generated by crossing VHL^fl/fl^ (012933, The Jackson Laboratory), VHL^fl/fl^ (Cramer, et al) and Arg‐1^fl/fl^ (008817, The Jackson Laboratory) with heterozygotic LysM^Cre+^ mice (004781, The Jackson Laboratory). All in vivo experimental procedures were performed in mice aged between 8 and 16 weeks of age, comparing heterozygotic LysM^Cre+^ with floxed littermates, and approved by the local animal ethic board (Stockholm north, N101/16). Housing and care of research animals was done according to Swedish national guidelines.

### 4.2 Bone marrow‐derived macrophages

To obtain BMDM, bone marrow cells were collected from murine tibia and femur, cultured for 7 days in DMEM (11995065, Gibco^™^) supplemented with 10% FBS (10270106, Gibco^™^), 1% penicillin/streptomycin (10378016, Gibco^™^), 10 ng/mL M‐CSF and GM‐CSF (416‐ML‐050/CF and 415‐ML‐050/CF, R&D Systems). Mature BMDMs were re‐plated and incubated in either room oxygen levels or in 1% oxygen for 6, 24 and 48 hours.

### 4.3 Lung fluid mass

Pulmonary oedema was established by determining the total fluid mass using the difference between wet and dry lung weights. Lungs were collected by dissection of deeply anaesthetized mice and weighted in order to determine the wet weight. For determination of the dry weight, the lungs were dried in 60°C incubator for 3 days and weighted. The difference between wet and dry lungs were defined as total water mass.

### 4.4 Transmission electron microscopy (TEM)

The lung tissues were fixed by vascular perfusion using in 2.5% glutaraldehyde + 1% paraformaldehyde in 0.1 mol/L phosphate buffer, pH 7.4 at room temperature and stored at 4°C. Specimens were rinsed in 0.1 mol/L phosphate buffer, pH 7.4 and postfixed in 2% osmium tetroxide 0.1 mol/L phosphate buffer, pH 7.4 at 4°C for 2 hours, dehydrated in ethanol followed by acetone and embedded in LX‐112 (Ladd). Semi‐thin sections (0.5 µm) were cut and stained with toluidine blue O and used for light microscopic analysis. Ultrathin sections (approximately 50‐60 nm) were cut by a Leica EM UC 6 (Leica) and contrasted with uranyl acetate followed by lead citrate and examined in a Hitachi HT 7700 at 80 kV. Digital images were taken using a Veleta camera (Olympus Soft Imaging Solutions, GmbH).

### 4.5 Pulmonary permeability

Pulmonary permeability was established by injecting the mice by the tail vein with 100 μL of 1% Evans blue (E2129, Sigma) in PBS. Euthanasia was performed 1‐hour later by Avertin (T48402, Sigma‐Aldrich) overdose (40 mg/mouse). BAL was collected by intra tracheal injection of 1 mL PBS (14190‐144, Gibco) and retreating the fluid shortly after the injection. The procedure was then followed by collection of blood through heart puncture. Permeability level was then determined by measurement of the levels of Evans Blue in BAL at absorbance of 620 nm.

### 4.6 Real time PCR

Lungs were collected from deeply anaesthetized mice and instantly frozen with liquid nitrogen. Tissue processing and RNA isolation were performed using Qiagen RNeasy Kit (cat nr: 74106) following the manufacturer's instructions. Later, cDNA was produced using iScript^™^ cDNA Synthesis Kit (1708890, Bio‐Rad) following the manufacturer's instructions. All primers used were predesigned and ordered from KiCqStart^®^ (Sigma‐Aldrich), gene ID, primer pair or sequence are demonstrated in Table 1.

**TABLE 1:** Primers used in real‐time PCR

Name	Gene name	Gene ID	Pair/sequence
4‐1BB	*Tnfrsf9*	21942	3
A20	*Tnfaip3*	21929	1
Abp1	*Abp1*	76507	3
Arg‐1	*Arg1*	11846	1
ARG2	*Arg2*	11847	1
ASMA	*Acta2*	11475	1
BAX	*Bax*	12028	1
Bcl2	*Bcl2l1*	12043	1
Birc5	*Birc5*	11799	1
BxL	*Bcl2l1*	12048	1
CCL4	*Ccl4*	20303	1
CD11b	*Itgam*	16409	1
CD11c	*Itgax*	16411	1
CD207	*Cd207*	246278	1
CD23	*Fcer2a*	14128	1
CD27	*Cd27*	21940	1
CD28	*Cd28*	12487	1
CD4	*Cd4*	12504	1
CD44	*Cd44*	12505	1
CD74	*Cd74*	16149	1
CD8a	*Cd8a*	12525	1
CD8b	*Cd8b*	12526	1
COL 1	*Col1a1*	12842	1
CSF1	*Csf1*	12977	1
CSF1R	*Csf1r*	12978	1
CSF2	*Csf2*	12981	1
CSF2R	*Csf2r*	12982	1
CTLA4	*Ctla4*	12477	1
CXCL2	*Cxcl2*	20310	1
F4/80	*Adgre1*	13733	2
FIH	*Hif1an*	319594	1
FIZZ1	*Retnla*	57262	1
Granzyme B	***Gzmb***	14939	1
HIF1	*Hif1a*	15251	3
HIF2	*Epas1*	13819	3
HK1	*Hk1*	15275	2
HPRT	*Hprt1*	15452	FW: TTATCAGACTGAAGAGCTACTGTAATGATC RW: TTACCAGTGTCAATTATATCTTCAACAATC
ICAM1	*Icam1*	15894	1
ICAM2	*Icam2*	15896	1
ICOS	*Icos*	54167	1
IL‐1b	*Il1b*	16176	1
IL‐6	*Il6*	16193	1
IL2r	*Il2rg*	16186	1
IL7r	*Il7r*	16197	1
iNOS	*Nos2*	18126	2
Lag3	*Lag3*	16768	1
Lamp3	*Lamp3*	239739	1
MHC Class II	*H2ab1*	14961	1
MRC1	*Mrc1*	17533	1
Ni	*Nfkbia*	18035	1
Nos3	*Nos3*	18127	1
OAT	*Oat*	18242	2
ODC	*Odc1*	18263	1
P4HA	*P4ha1*	18451	1
PD‐1	*Pdcd1*	18566	1
PDK	*Pdk1*	228026	1
PFK	*Pfkl*	18641	1
PGK	*Pgk2*	18663	1
PHD1	*Egln2*	112406	1
PHD2	*Egln1*	112405	1
PHD3	*Egln3*	112407	1
Plau	*Plau*	18792	1
Ptgs2	*Ptgs2*	19225	1
S100	*S100b*	20203	1
Sat1	*Sat1*	20229	1
Sell	*Sell*	20343	1
Srm	*Srm*	20810	1
TNFa	*Tnf*	21926	1
VCAM1	*Vcam1*	22329	1
VEGF	*Vegfa*	22339	1

Note

Name, gene name, Gene ID and primer pair or sequence are presented for all primer pairs used in this study.

### 4.7 Western blot

Tissues for Western blot were immediately frozen using liquid nitrogen, homogenized with mortar and pestle and resuspended in 1 mL RIPA buffer (R0278, Sigma‐Aldrich) containing cOmplete^™^ Protease Inhibitor Cocktail (11697498001, Roche). Nuclear protein extraction from tissue cultures were obtained using NE‐PER^™^, according to the manufacturer's protocol. Protein extract concentrations were determined using Pierce^™^ BCA Protein Assay Kit (23225, Thermo Fisher) then separated by NuPAGE^™^ 4%‐12% Bis‐Tris Protein gel (NP0336BOX, Thermo Fisher) and transferred to nitrocellulose membranes (1704158, Bio‐Rad) using Trans‐Blot Turbo Transfer System (Bio‐Rad Laboratories, Inc). All steps in this procedure were performed according to the manufacturer's instructions. Prior to antibody staining, nitrocellulose membranes were then blocked for 1 hour in Roti^®^‐Block (A151, Carl Roth) diluted in water.

Primary antibody staining was done using rabbit anti Arg‐1 antibody (D4E3M,CST), rabbit anti HIF1a (GTX127309, GeneTex), rabbit anti HIF2a (NB100‐122, Novus Biologicals) and mouse anti Lamin B1 antibody (sc‐374015, Santa Cruz) incubated overnight diluted to 1:1000 and 1:500 respectively. For secondary antibody staining the Goat anti rabbit 800CW (926‐32211, Li‐Cor) and Goat anti mouse 680RD (926‐68070, Li‐Cor) 1 hour in RT, all antibodies were diluted in Roti^®^‐Block and 0.1% Tween (P1379, Sigma‐Aldrich).

### 4.8 Histological assessment

Mice were euthanatized by avertin overdoes, then alveolar space was perfused with 4% Formaldehyde solution (252549, Sigma‐Aldrich) in PBS (14190‐144, Gibco), PFA, for 2 minutes prior to dissection. The whole lungs were then incubated in PFA for 24 hours followed by incubation in 70% ethanol (01370, Histolab). Post dehydration the lungs were imbedded in paraffin and sectioned at 5 μm thickness. Tissue slides were fixated on the glass by incubation in 60°C for 45 minutes. This was followed by removal of paraffin with 3× 5 minutes incubation in Histoclear (14250, Histolab) and rehydration of sectioned tissue by 2× 5 minutes incubation in 99% ethanol (01399, Histolab), 2× 5 minutes incubation in 96% ethanol (01396, Histolab), 2× 5 minutes incubation in 70% ethanol (01370, Histolab), 2× 5 minutes incubation in H_2_O and finally 5 minutes incubation in PBS. Tissue staining for arginase‐1 was performed using anti Arg‐1 antibody (D4E3M, CST) followed by secondary antibody incubation with Anti‐Rabbit IgG (B8895, Sigma‐Aldrich) diluted 200 times. Immunohistochemical staining was then developed using peroxidase complex VECTASTAIN^®^ Elite^®^ ABC HRP Kit (PK‐6100, Vector Laboratories) and DAB Peroxidase (HRP) Substrate Kit (SK‐4100, Vector Laboratories), all according to the manufacturer's instructions.

### 4.9 Tissue immunofluorescence assessment

Lung tissue was frozen with liquid nitrogen and embedded in Optimal cutting temperature compound (4583, Sakura) and sectioned at 10 μm thickness, followed by immediate fixation in 96% ice‐cold ethanol (01396, Histolab) for 30 minutes followed by 3× 5 minute washing in PBS (14190‐144, Gibco). Tissue staining was then performed using BlockAid^™^ (B10710, Invitrogen), followed by overnight incubation in 4°C with primary antibodies against HIF1a (ab82832, abcam) or HIF2a (NB100‐122, Novus) diluted 100 times. Detection was performed by staining with anti‐Rabbit IgG Alexa Fluor Plus 680 (A32802, Invitrogen) at room temperature for 1 hour followed by washing with PBS and mounting with ECTASHIELD^®^ Antifade Mounting Medium with DAPI (H1200, Vector Laboratories).

### 4.10 Flow cytometry

Flowcytometry was performed using same materials and methods as described earlier.^21^ Pulmonary single cell suspension was prepared using Lung dissociation Kit (130‐095‐927, Miltenyi Biotec) according to manufacturer instructions. The single‐cell suspension was then used for two different staining panels targeting myeloid or lymphoid populations. Viability staining was performed using Aqua Dead Cell Stain Kit (L34965, Thermo Fisher) or Near‐IR Dead Cell Stain Kit (L34975, Thermo Fisher) according to the manufacturer's instructions, followed by 10‐minute FC blocking. Myeloid or Lymphoid antibody mixes (Table 2) were applied for 30 minutes in 4°C prior to flow cytometry analysis. Figures S6 and S7 demonstrate gating strategies used in this study.

**TABLE 2:** Stains and antibodies used to stain single‐cell suspensions in flow cytometry

Stain name	Antigen	Fluorochrome	Dilution	Clone
Lymphoid	Live/dead	APC‐Cy7	500	
	Fc block		50	93
	CD45.2	Brilliant Violet 421	200	104
	CD8	Brilliant Violet 510	400	53‐6,7
	B220	Alexa Fluor 488	200	RA3‐6B2
	GD TCR	PE	200	GL3
	CD3	PerCP/Cy5.5	100	17A2
	CD4	PE‐Cy7	400	GK1.6
	NK1.1	APC	286	PK136
Myeloid	Live/dead	Aqua	500	
	Fc block		50	93
	CD11b	Horizon V450	200	M1/70
	NK1.1	Alexa Fluor 488	200	RA3‐6B2
	B220	Alexa Fluor 488	200	PK136
	Siglec‐F	PE	200	E50‐2440
	Ly6G	PerCP/Cy5.5	500	1A8
	CD11c	PE‐Cy7	125	HL3
	CD45.2	APC	200	104
	Ly6C	APC‐H7	500	AL‐21

### 4.11 Physiological tests

Voluntary running data were established by housing two female mice in cages with free access to running wheels (Med Associates Inc ENV‐044) with wireless activity tracking features. Distance, speed and active periods were monitored using software provided by the manufacturer (Med Associates Inc SOF‐860 Wheel Manager and Med Associates Inc SOF‐861 Wheel Analysis). After 4 days of acclimatization, the running activity in mice was traced for 4 weeks.

Echocardiography was performed in anaesthetized male mice using Vevo^®^ 1100 (VisualSonics^®^). Blood pressure values were acquired with CODA 4‐channel High Throughput Non‐Invasive Blood Pressure System (Kent Scientific Corporation). A 4‐day training was performed before the final values were documented. ECG values were collected in a freely moving recording platform, ECGenie (Mouse Specifics), all measurement took place in the afternoon. Indirect Open circuit calorimetry was performed by caging mice in Somedic INCA (Somedic AB) after 2 hours of acclimatization period for 22 hours.

Methacholine and hypoxia challenge were performed using 4 chamber Whole Body Plethysmograph (Emka Technologies) with 15 minutes acclimatization prior start of the experiment. All procedures were performed according to the manufacturer's protocol.

### 4.12 RNA sequencing

Lungs were collected from 11‐weeks‐old female mice euthanized by avertin overdose. Tissue lysis and RNA isolation were performed with liquid nitrogen and RNeasy Plus mini Kit (74136, QIAGEN) according to the manufacturer's protocol. Total RNA was subjected to quality control with Agilent Tapestation according to the manufacturer's instructions. cDNA synthesis and RNA sequencing was performed using Illumina Nextseq 550 (Illumina, Inc) and TruSeq Stranded RNA (770‐2012‐010‐D, Illumina) following the manufacturer's protocol. Evaluation of raw sequencing data was done with FAST‐QC and basic gene mapping was done using mouse reference genome. Differential analysis was performed using limma‐voom algorithm with false discovery rate cutoff at 0.05. Pathway enrichment analysis and other statistical analysis was performed on iDEP.90 platform.^25^

## CONFLICT OF INTEREST

The authors declare that the research was performed without any commercial or financial conflict of interest.

## AUTHORS CONTRIBUTIONS

MG, PV, HR and RSJ designed and interpreted the experiments in this study. MG, GSD, PV and HR were involved in data collection. MG, RSJ and HR wrote the manuscript. All authors reviewed and approved the final manuscript. RSJ supervised the work.

## ETHICS STATEMENT

Animal handling and health monitoring was performed according to the national guidelines for animal care and research. All experiments in this study have been reviewed and approved by the Stockholm north ethical committee (N101/16).

## Supporting information

Figs S1‐S9

## Data Availability

The data that support the findings of this study are available from the corresponding author upon reasonable request.
