# Supplementary material for: Deregulated hypoxic response in myeloid cells: A model for high‐altitude pulmonary oedema (HAPE)
Source: Acta Physiol (Oxf). 2020 Mar 16;229(2):e13461. doi: 10.1111/apha.13461 (PMC8638671; doi:10.1111/apha.13461)
Supplement: Supplementary file 1 — Figs S1‐S9 [file APHA-229-e13461-s001.docx]

**Supplementary Figure 1 Deleted myeloid VHL results in upregulation of HIF1a and HIF2a.** Immunofluorescence staining of frozen lung sections for (A) HIF1a and (B) HIF2a and western blot for HIF1a and HIF2a in nuclear extractions of bone marrow derived myeloid cells, HEK293 cells overexpressing degradation- resistant HIF1a and HIF2a were used as positive control. n = 3 mice per group for immunofluorescence staining, aged between 10 - 16 weeks.

**Supplementary Figure 2 Immunological composition of pulmonary myeloid populations.** Comparison of cell numbers in whole lung single cell suspension A) alveolar macrophages as CD11c^+^ Siglec F^+^, B) eosinophils as CD11c^-^ Siglec F^-^, C) neutrophils as Ly6G^+^ , D) monocytes as Ly6c^+^, and mononuclear myeloid cells as E) with high CD11b expression (CD11b^hi^ CD11c^+^) and F) intermediate expression (CD11b^INT^ CD11c^-^) followed by G) gating strategy used in this experiment where gates with arrows show the populations used for the following gating.

Data presented as Tukey boxplots, * = P< 0.05, † = P< 0.01, ‡ = P<0.001, ns = not significant. Statistical analysis was performed with unpaired T test, n= 4-5 mice per group aged between 10 - 16 weeks.

**Supplementary Figure 3 Immunological composition of Bronchioalveolar lavage.** Comparison of cell frequencies and numbers in whole bronchioalveolar lavage. Frequencies of A) neutrophils as Ly6G^+^, B) monocytes as Ly6C^+^, C) mononuclear myeloid cells as CD11c^+^ and D-F their represented total number in BAL followed by G) protein concentration in BAL fluid and H) gating strategy used in this experiment where gates with arrows show the populations used for the following gating.

Data presented as Tukey boxplots, * = P< 0.05, † = P< 0.01, ‡ = P<0.001, ns = not significant. Statistical analysis was performed with unpaired T test, n= 4-5 mice per group aged between 10 - 16 weeks.

**Supplementary Figure 4 Deletion of myeloid VHL results in increased lung to body weight ratio**. Lungs where collected from wild type floxed mice (VHL^fl/fl^) and mice lacking myeloid VHL (VHL^fl/fl^ LysM^Cre+^) aged between 10 - 12 weeks. (A) Body weight of mice used in the experiment and (B) lung to body weight ratio.

Data presented as Tukey boxplots, * = P< 0.05, † = P< 0.01, ‡ = P<0.001, ns = not significant. Statistical analysis was performed with unpaired T test, n= 7-8 mice per group

**Supplementary figure 5 Blood pressure measurements and ECG shows no signs of any cardiological dysfunctions**.

Tail cuff blood measurements in resting mice of A) Heart rate, B) Mean arterial pressure (MAP), C) diastolic, D) systolic pressure and E) flow rate.

ECG values where measured on free moving mice F) Heart rate (HR), G) RR, H) PQ, I) PR, J) QRS, K) QT, L) ST, M) QTC, N) Mean SR AMP, O) Mean R Amp, P) QT dispersion, Q) QTc Dispersion. Data presented as Tukey boxplots, * = P< 0.05, † = P< 0.01, ‡ = P<0.001, ns = not significant. Statistical analysis was performed with unpaired T test, n= 8 male mice per group aged between 10 - 16 weeks.

**Supplementary figure 6 Echocardiography and Indirect Open circuit calorimetry of VHL^df/df^ LysM^cre+^ mice and circularly parameters**

Echocardiography data on anesthetized mice of the left ventricle in SAX-M mode showing A) diastolic end volume, B) systolic end volume, C) left ventricular mass, D) Ejection Fraction, E) Fractional Shortening.

Mice were housed in INCA system for 24 hours measuring F) O^2^ consumption and G) CO^2^ production for 22 hours post 2 hours acclimatization period. Shaded aria represents the night cycle. Data presented as Mean and Standard deviation over time, * = P< 0.05, † = P< 0.01, ‡ = P<0.001, ns = not significant. Statistical analysis was performed with two way ANOVA, n= 8 male mice per group aged between 10 - 16 weeks.

**Supplementary figure 7 Methacholine challenge of VHL^df/df^ and VHL^df/df^ LysM^cre+^ mice** **do not show any asthmatic phenotype.**

Methacholine (MCh) challenge was preformed using 4 chamber Whole Body Plethysmography for mice, with increasing concentrations as demonstrated on X axis. A) Enhanced Pause (Penh) a Dimensionless indicator of broncho-constriction, computed as: (TE/RT – 1) * (PEF/PIF), B) Inspiratory Time (Ti) Time from start of inspiration to end of inspiration, C) Expiratory Time (Te) Time from start of expiration to beginning of next inspiration, D) Peak Inspiratory Flow (PIF) The maximum negative flow during one breath, E) Peak Expiratory Flow (PEF) the maximum positive flow during one breath, F) Tidal Volume (TV) the integral of the flow during inspiration (negative period), G) Expired Volume (EV) the integral of the flow during expiration (positive period), H) Minute Volume (MV) the total volume breathed over one minute based on current breathing rate: it is computed on a breath-by-breath basis (TV*F), I) Frequency of breathing, the breath-by-breath rate of breathing, computed from PEF to PEF or TI+TE.

Data presented as Mean and Standard deviation over time, * = P< 0.05, † = P< 0.01, ‡ = P<0.001, ns = not significant. Statistical analysis was performed with two way ANOVA, n= 8 male mice per group aged between 10 - 16 weeks.

**Supplementary figure 8 Hypoxia challenge of VHL^df/df^ and VHL^df/df^ LysM^cre+^ mice**.

Hypoxia challenge was preformed using 4 chamber Whole Body Plethysmography for mice, with 10% O_2_ for 10 minutes shown as shaded area in each graph A) Enhanced Pause (Penh) a Dimensionless indicator of broncho-constriction, computed as: (TE/RT – 1) * (PEF/PIF), B) Inspiratory Time (Ti) Time from start of inspiration to end of inspiration, C) Expiratory Time (Te) Time from start of expiration to beginning of next inspiration, D) Peak Inspiratory Flow (PIF) The maximum negative flow during one breath, E) Peak Expiratory Flow (PEF) the maximum positive flow during one breath, F) Tidal Volume (TV) the integral of the flow during inspiration (negative period), G) Expired Volume (EV) the integral of the flow during expiration (positive period), H) Minute Volume (MV) the total volume breathed over one minute based on current breathing rate: it is computed on a breath-by-breath basis (TV*F), I) Frequency of breathing, the breath-by-breath rate of breathing, computed from PEF to PEF or TI+TE.

Data presented as Mean and Standard deviation over time, * = P< 0.05, † = P< 0.01, ‡ = P<0.001, ns = not significant. Statistical analysis was performed with two way ANOVA, n= 8 male mice per group aged between 10 - 16 weeks.

**Supplementary figure 9 Deleted myeloid VHL results in upregulation of 392 genes and downregulation of 44 genes.** RNA sequencing preformed on murine female lungs at age of 11 weeks. A) Principal component test B) Volcano plot showing log2fold change in VHL^fl/fl^ LysM^Cre^ mice comparted to VHL^fl/fl^, Y axis shows the adjusted p valye, the line stands for significance. C) Top 10 most significant GO pathway enrichment and D) ten most downregulated and upregulated genes in VHL^fl/fl^ LysM^Cre^ mice comparted to VHL^fl/fl^. N=4, adjusted p values were calculated by false discovery rate test.
